# Brain Injury and Dementia in Pakistan: Current Perspectives

**DOI:** 10.3389/fneur.2020.00299

**Published:** 2020-04-30

**Authors:** Maheen M. Adamson, Sadia Shakil, Tajwar Sultana, Muhammad Abul Hasan, Fatima Mubarak, Syed Ather Enam, Muhammad A. Parvaz, Adeel Razi

**Affiliations:** ^1^Department of Neurosurgery, Stanford University School of Medicine, Palo Alto, CA, United States; ^2^Department of Rehabilitation, VA Palo Alto, Palo Alto, CA, United States; ^3^Department of Electrical Engineering, Institute of Space Technology, Islamabad, Pakistan; ^4^Turner Institute for Brain and Mental Health, and Monash Biomedical Imaging, Monash University, Clayton, VIC, Australia; ^5^Department of Biomedical Engineering, NED University of Engineering and Technology, Karachi, Pakistan; ^6^Neurocomputation Laboratory, National Centre for Artificial Intelligence, NED University of Engineering and Technology, Karachi, Pakistan; ^7^Department of Computer and Information Systems Engineering, NED University of Engineering and Technology, Karachi, Pakistan; ^8^Department of Radiology, Aga Khan University, Karachi, Pakistan; ^9^Department of Surgery, Aga Khan University, Karachi, Pakistan; ^10^Department of Psychiatry, Icahn School of Medicine at Mount Sinai, New York, NY, United States; ^11^Department of Neuroscience, Icahn School of Medicine at Mount Sinai, New York, NY, United States; ^12^The Wellcome Centre for Human Neuroimaging, Institute of Neurology, University College London, London, United Kingdom; ^13^Department of Electronic Engineering, NED University of Engineering and Technology, Karachi, Pakistan

**Keywords:** TBI, dementia, Alzheimer's disease, Pakistan, aging, road traffic accidents, violence

## Abstract

Alzheimer's disease (AD) is the most common form of dementia, accounting for 50–75% of all cases, with a greater proportion of individuals affected at older age range. A single moderate or severe traumatic brain injury (TBI) is associated with accelerated aging and increased risk for dementia. The fastest growth in the elderly population is taking place in China, Pakistan, and their south Asian neighbors. Current clinical assessments are based on data collected from Caucasian populations from wealthy backgrounds giving rise to a “diversity” crisis in brain research. Pakistan is a lower-middle income country (LMIC) with an estimated one million people living with dementia. Pakistan also has an amalgamation of risk factors that lead to brain injuries such as lack of road legislations, terrorism, political instability, and domestic and sexual violence. Here, we provide an initial and current assessment of the incidence and management of dementia and TBI in Pakistan. Our review demonstrates the lack of resources in terms of speciality trained clinician staff, medical equipment, research capabilities, educational endeavors, and general awareness in the fields of dementia and TBI. Pakistan also lacks state-of-the-art assessment of dementia and its risk factors, such as neuroimaging of brain injury and aging. We provide recommendations for improvement in this arena that include the recent creation of Pakistan Brain Injury Consortium (PBIC). This consortium will enhance international collaborative efforts leading to capacity building for innovative research, clinician and research training and developing databases to bring Pakistan into the international platform for dementia and TBI research.

## Introduction

Alzheimer's disease (AD) is the most common form of dementia, accounting for 50–75% of all cases, with a greater proportion of individuals affected at older age range. A single moderate or severe traumatic brain injury (TBI) is associated with accelerated aging and increased risk for dementia (1–3). The 2014 Alzheimer's Association Facts and figures Guidelines include TBI as a risk factor for AD along with advanced age, sex, family history of AD, positive Apo-e4 allele, cardiovascular disease, social, and cognitive engagement and education (4). Currently, 58% of the world's population aging with dementia live in low-middle income countries, but by 2050 this will rise to 68%. The fastest growth in the elderly population is taking place in China, Pakistan, and their south Asian and western Pacific neighbors (5). However, current clinical assessments are based on data collected from Caucasian populations from relatively wealthy backgrounds (3, 6–9), giving rise to a “diversity” crisis in brain research. This lack of ethnic diversity means that: (1) there is a lack of data that could teach us how AD progresses in populations with distinct backgrounds, especially in terms of political conflict, violent crime (including domestic and gender-based violence) and environmental hazards, and (2) there is little understanding about the predictors of brain or mental health that can be generalized from Caucasians to other ethnic groups. In this paper, we provide an overview of TBI and dementia incidence, management, and services available in a low-middle income country such as Pakistan. We also specifically point to a dire need of neuroscientific research in both TBI and AD in Pakistan. We conclude by presenting our recent initiative, Pakistan Brain Injury Consortium (PBIC), which seeks to improve the research and clinical services in Pakistan by bringing in international expertise, promoting research into TBI causes and treatment, training of staff, and creating awareness around TBI importance and care.

## Traumatic Brain Injury and Dementia

A TBI occurs when the head is injured by a blow or penetration of an object resulting in brain damage. These injuries can be due to falls, road traffic accidents, athletic activities, firearms accidents, or assaults. Based on severity, TBI can be categorized as mild, moderate, or severe. Mild TBI would mostly result in concussions that are temporary and not life threatening while severe TBI may result in long periods of unconsciousness, coma, or even death. Although reports of mild TBI patients returning to baseline (pre-injury) functioning 1 year post-injury have been documented, 7–33% of these patients experience persistent symptoms (10). TBI occurs frequently in young people and is the most common cause of disability and death between the ages 1 and 45 (11). Annually, 10 million people are affected by TBI (11) and based on American Association of Neurological Surgeons about 1.7 million cases of TBI occur only in the US every year and 5.3 million people live with disabilities caused by TBI in the US, alone (12). The majority of those with TBI will recover in a matter of weeks (13). However, 10–30% will experience a persistent set of symptoms lasting for months, even years (14–16). Cognitive, sensory, and affective complaints erode the quality of life for these patients (17, 18), and such sequelae has been collectively labeled as persistent post-concussion syndrome (19–21).

A single blow to the head classified as a moderate or severe TBI is associated with progressive cognitive decline leading to dementia [reviewed in (1, 2)]. The recent attention given to Chronic Traumatic Encephalopathy (CTE; formerly known as dementia pugilistica) encountered in military personnel, veterans and in those who participate in contact sports, has raised much public concern. These links are particularly disturbing because they are associated with many alarming features. The rate of recovery from “mild” TBI is likely to be different for older adults and perhaps also impacting their quality of life, which may be much different in younger patients. Co-morbid conditions such as post-traumatic stress disorder (PTSD) and depression may prolong the chronic symptoms of TBI resulting in cognitive decline and dementia (22). Populations with TBI in areas with sociopolitical conflict (such as blast victims during suicide bombing and political riots), in addition to military personnel and veterans, might be at greater risk for accelerated cognitive decline and dementia as they grow older.

Jordan (23) provides an extensive review of the spectrum of chronic traumatic brain injury in sports, of which the most clinically pertinent are CTE, chronic post-concussion syndrome, and chronic neurocognitive impairment (NCI). Briefly, CTE represents the long-term neurologic consequences of repetitive mild TBI and is secondary to progressive tauopathy (24, 25). There are several risk factors associated with the development of CTE but exposure to contact sports is currently the most validated one. Dementia-like cognitive difficulties are commonly observed as the disease progresses and the pathophysiology of CTE is also well-defined. Chronic post-concussion syndrome is clinically distinct from CTE and has an acute onset related to the single TBI event. However, the most pertinent chronic TBI sequelae to the development of age-related dementia is what Jordan (23) classifies as chronic NCI. Although vague, it encompasses a large variety of symptoms that are a result of sports-related (perhaps blast-related as well) repetitive TBI. The symptoms may manifest within a year or even years after the event. It has no established relationship with CTE and can be described by neuropsychological testing (26). Most studies compared the performance in patients with NCI with either healthy controls or with performance prior to the event to ascertain decrements in performance. Specifically, impaired episodic memory has been reported in Jockeys after injury (27), and boxers with APOE ε4 allele have more neurological impairment than those without (28). Perhaps most direct evidence comes from McAllister et al. (29), who reported deficits in new learning, verbal learning, and memory in collegiate contact sport athletes at post- compared to pre-season. Cognitive deficits such as naming and word finding in visual/verbal episodic tasks are also documented in aging retired National Football League (NFL) players, and such deficits were correlated with white matter abnormalities on MRI (30). Volumetric MRI abnormalities, particularly reduced volume in caudate, hippocampus, and amygdala, have been reported in boxers and mixed martial artists (31). Interestingly, Singh et al. (32) reported reduced hippocampal volumes in football players compared to healthy controls that were inversely correlated with football exposure. Reduced glucose metabolism was also observed in positron emission tomography (PET) scans in posterior cingulate cortex, parieto occipital lobes, frontal lobes, and cerebellum in retired boxers (33). Despite growing neuroimaging evidence, current TBI guidelines in the US do not recommend imaging as a diagnostic tool at the acute stage of mild and moderate TBI. Nevertheless, most diagnostic and treatment studies of TBI and dementia done in developed countries regularly utilize neuroimaging techniques.

Such a trend, albeit important to understanding the neurobiology of TBI and its association with cognitive decline, places developing countries at a disadvantage due to the lack of cutting-edge neuroimaging and clinical resources. Indeed, the prevalence of TBI and resulting psychiatric complications and disabilities constitute a huge burden on the economy and resources in developing countries like Pakistan. Hence, the goal of this paper is to present the current status of TBI and dementia incidence and the management of these health problems in Pakistan.

## Incidence of Brain Injury and Management in Pakistan

**Figure 1:**
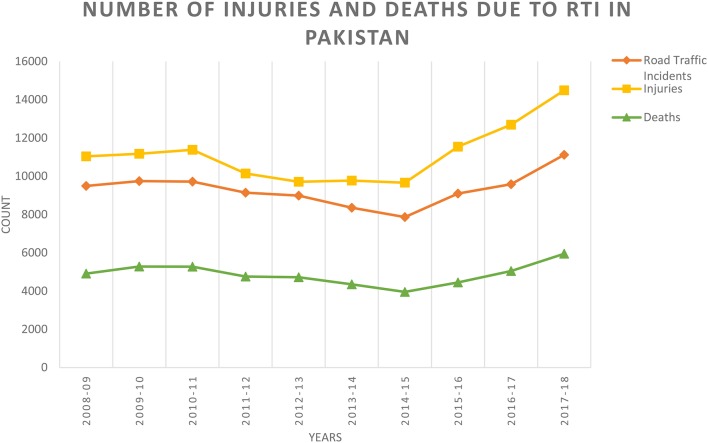
Number of injuries and deaths due to Road Traffic Incidents (RTI) in Pakistan (35). Date represents information taken from: http://www.pbs.gov.pk/sites/default/files/tables/Traffic%20Accidents%20%28YEARLY%29.pdf.

**Figure 2:**
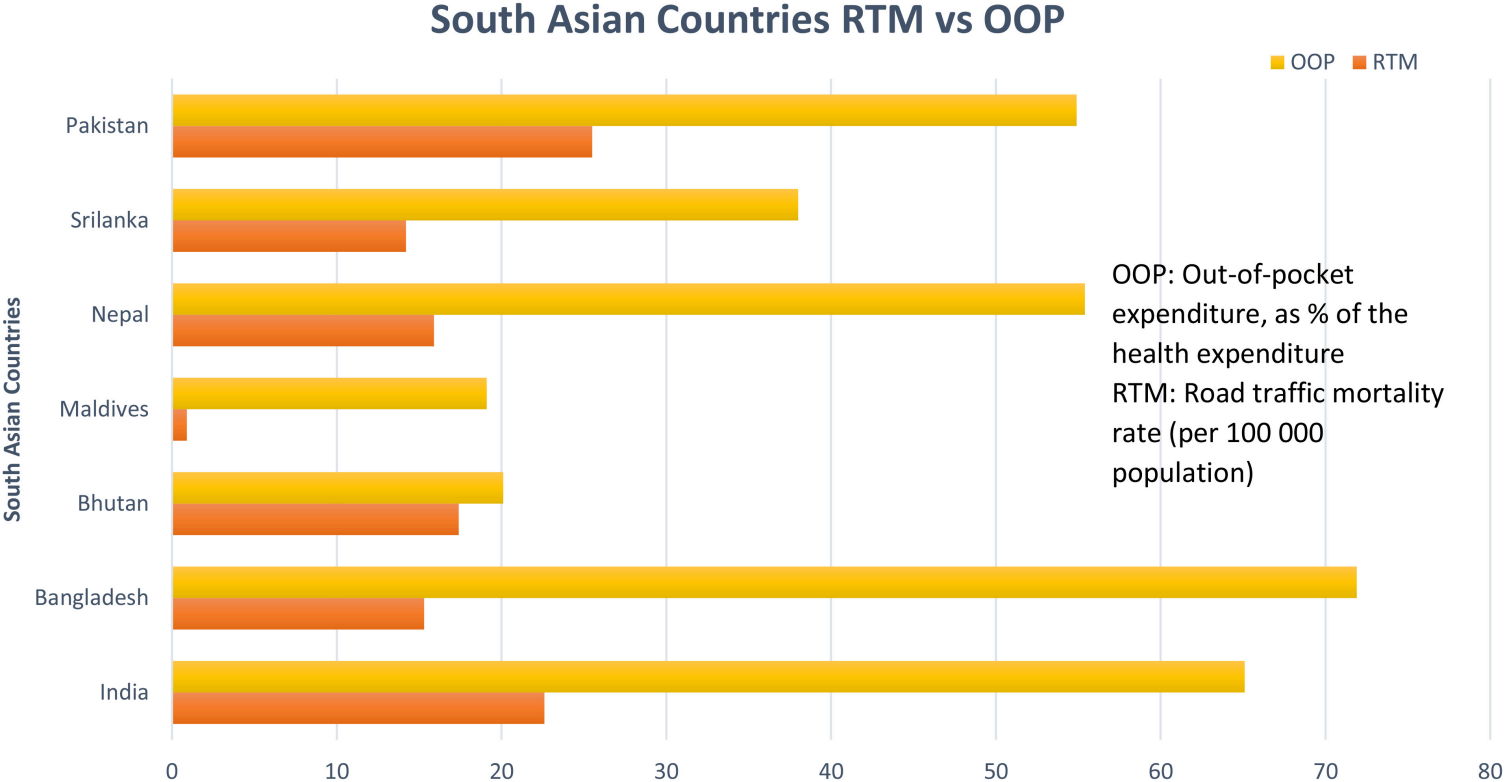
Road Traffic Mortalities (RTM) rates and Out-of-pocket Expenditure across South Asia. OOP, Out-Of-Pocket (OOP) expenditure, as % of the health expenditure; RTM, Road Traffic Mortality rate (per 100,000 population). Data reported from Pakistan is from 2012 and for the rest of the countries is from 2016 [Data Source: World Health Organization (WHO)] (44).

**Figure 3:**
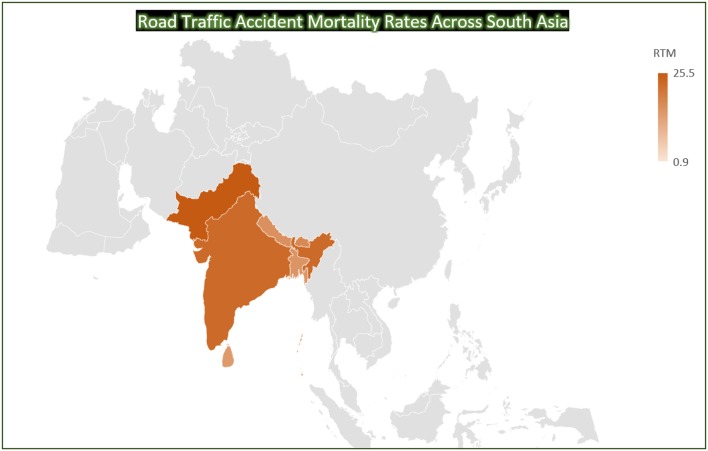
Road Traffic Accident Mortality Rates Across South Asia. Road Traffic Mortality rate (RTM) per 100,000 population (44) (Map Template Courtesy: Bing).

Razzak et al. (34) conducted a systematic review of brain injury incidence and risks in Pakistan from the perspective of a low-middle income country (34). In this review, they pointed out that The Eastern Mediterranean region of the World Health Organization, which includes Pakistan, has some of the highest death rates from injuries such as Road Traffic Incidents (Figure 1) and political conflicts [146,000 deaths and 2.8 million injuries just from road traffic crashes (36, 37)] (Table 1). Figures 2, 3 depict the Road Traffic Mortality Rates (RTM) across South Asian countries, with Pakistan reporting highest RTM at 25.5 per 100,000 population, even higher than India (44). Injuries caused 42 deaths per 100,000 population or 6% of all deaths (45). Specifically, injuries contribute about 11% of all deaths above the age of 60 years; 57% of all injuries occur among 15–59 year olds, with males (8 vs. 4% females) being more likely to suffer injuries (46, 47). Risk factors also include the lack of organized prehospital and hospital based trauma care (48). Additionally, domestic violence against women is a significant problem in Pakistan; the rates of physical and sexual violence estimated to be as high as 80 and 77%, respectively (42). In terms of political conflict, Pakistan is among the world's top five countries most affected by terrorism (49). The country had over 12,000 terrorist attacks between 2009 and 2016, resulting in 16,526 deaths. Suicide terrorist events, where civilians are victims in almost all events, make up 74.1% of deaths and 93.8% of those injured (49). Terrorist attacks and violent clashes among different political parties during the 2013 general elections led to 298 deaths and 885 injuries between January 1 and May 15 of that year alone (48). The province of Sindh—primarily Karachi was the most affected by both terrorist attacks and incidents of political violence (34). Between January and April, 2018, Lady Reading hospital Peshawar admitted an average of 281 patients per month—a city prone to Taliban violence (43). Although acute care is readily available via excellent ambulance networks (34, 39), there are no follow-up rehabilitation services available. Additionally, there is no current patient registry for follow-up, biobank or any infrastructure available to provide care and education to the aging population, compounding the long-term burden of TBI.

**Table 1:** Brain injuries and deaths due to TBI in Pakistan.

**Year/Duration of data collection**	**Data source**	**Brain injuries**	**Deaths**	**Mechanism(s)**	**Reference(s)**
1990–1993	Neurosurgical units in Karachi and Quetta	100	52	Missile Injuries (gunshot and ballistic missiles)	(38)
1993–1996	Edhi ambulance transportation, Karachi	4,091	2,400	Violence	(39)
1995–1999	Neurotrauma Centers in different areas all over the country	260,000	46,800	Road traffic accidents, Fall from Height, Assault, Agriculture Injuries, Sports Injuries, Fall from Train	(40)
2007–2011	Combined Military Hospital, Quetta	1,056	83	Road traffic accidents, falls, gunshot wounds, social violence, bomb blast, sports related, mine blast, splinter injury	(41)
2013	Global Terrorism Index, 2016	885	298	Terrorist attacks and violent clashes	(42)
2018 (Jan–Apr)	Lady Reading Hospital, Peshawar	1,125	–	Multiple	(43)

Similar to the US, children and youth are the age groups most affected by the TBI in Pakistan also (40, 41, 50, 51). The major cause of TBI in youth is road traffic accidents (40, 41); while in children, it is falls from a certain height (50, 51). Another rapidly increasing cause of TBI in Pakistan is the penetrating brain injuries (PBIs) occurring due to gunshots or other firearms (38, 52–54). The increase in such injuries is attributed mainly with the increase in level of violence and terrorism due to bomb blasts and suicidal bomb attacks (54) (Note: We expect this rate of PBI occurrence to have slowed following much improved security condition since 2016, however, no recent formal study has been published to provide such statistics), These injuries are broadly divided into three categories (52); (1) Tangential with gunshots glancing off the skull without entering, (2) Penetrating with gunshots entering the skull, and (3) Perforating with gunshot entering and exiting the skull. Penetrating and perforating injury patients are at a higher risk of mortality compared to tangential injury patients (51). PBI patients need surgeries more than other TBI patients and lack of neurosurgical facilities and neurosurgeons increase the mortality rate of PBI (52) patients. Management of PBI differs a lot from the non-penetrating brain injury and “Guidelines for Management of Penetrating Brain Injury” is very useful to handle such injuries (53). Sports related head injuries, especially because of the most popular sport of Cricket in Pakistan, are also common and can be fatal (55). However, not many sports related cases are reported simply because of the lack of awareness about them in the society (56).

In addition to the damage caused by TBI to the brain immediately or within a few days after the injury, there are chances of developing neurological issues and psychiatric disorders (11). Pituitary dysfunction is also very common among TBI survivors with moderate and severe TBI, which may have neurological consequences and can result in increased morbidity (57). Modifications in molecular mechanism can also occur in TBI patients increasing the chances of epilepsy and Alzheimer's disease (58).

TBI cases are increasing rapidly in Pakistan with the increase in population, poor safety considerations, and frequent incidents of terrorism. However, available infrastructure for diagnosis, treatment provision, and follow-up care is not adequate. There are many reasons for this lack of facilities but the major reasons are lack of resources such as trained medical staff and state-of-the-art medical equipment. Based on a published report there were only 35 neurosurgical centers and only one neurosurgeon per 1.37 million people in Pakistan (52). Furthermore, even these centers do not have enough ambulances and trained paramedics to transport the TBI patients from the site of injury to these centers and to collect all the necessary information regarding any such injury. A multicenter TBI emergency care study reported out-of-pocket costs might be one of the major causes of TBI-based deaths or disabilities (59). Almost two-third of the population earned $2 a day per head in 2015 so affording the conveyance to the hospital (~$8) and subsequent CT scan (~$16) remained out of reach for many (59). In addition to the lack of resources, there is a lack of awareness about the importance of TBI patients' care immediately after the injury. Pakistani society also has unusual perceptions and sensitivities regarding the safety measures. For example, in Saeed et al. (60), the authors report that the female pillions, involved in TBI incidents, admitted not wearing helmets while riding a motorcycle and said that they would do so if they were male. Their reasons for not doing so was that they would look odd and societal pressure due to it being uncommon for females.

## Dementia and Alzheimer's Incidence and Current Management in Pakistan

Pakistan is the sixth most populated country in the world and currently has an estimated 150,000–200,000 patients with dementia (61). Life expectancy has increased in Pakistan in general leading to an increase in prevalence of dementia from 2 to 6% in persons older than 65 years of age (62). Treatment focuses on behavioral and caregiver issues and management of this older population poses an economic challenge. Alzheimer's disease International is leading a dementia registry with Shifa International hospital in Islamabad, and a roll-out of national dementia guidelines. In the absence of a geriatric medicine subspecialty in Pakistan, dementia care falls under neurology and psychiatry's domain. Khan et al. (52) provides alarming numbers: one dementia trained specialist, one dementia registry, one dementia research center, two academic research clinics, and one dementia day care center in the entire country of Pakistan—a country of 197 million people (63). Of the 1,086 AD clinical trials conducted across the world, none are being conducted in Pakistan (63). The solution to this paucity of clinical research and care infrastructure must include international collaborative efforts, training of clinicians, and researchers in sub-specialties of neurology and psychiatry, large longitudinal studies including clinical trials with genetic, biomarker and neuroimaging measures, and an effort to translate and validate psychological instruments (52, 61). Higher Education Commission (HEC), a national body that oversees all aspects of tertiary education and research in Pakistan, recognizes these needs and recommends deepening research collaboration across the globe and reforming research funding frameworks (64).

## Discussion

Our review demonstrates the lack of resources in terms of speciality trained clinician staff, medical equipment, research capabilities, educational endeavors, and general awareness in both the fields of dementia and TBI. In terms of clinical follow-up and existing registries, Pakistan National Emergency Departments Surveillance Study [Pak-NEDS (2010–2011); (59)] and Road Traffic Injury Research and Prevention Center [RTIRPC (2007–2017); (65)], are two databases developed previously. However, both databases are no longer collecting data but are available for recruitment. There are currently no biobanks available for brain injury or dementia in Pakistan either. Despite the state-of-the-art clinical research facilities available at the top ranking hospitals, such as AKU, these biobanks need to be developed, with database protocols and an emphasis on clinical and research staff training and community outreach.

In order to mitigate the road safety immediate concerns, the Sindh Governor Road Safety Advisory Board recommended the formation of National Road Safety Council (NRSC) to establish the National Road Safety Action Plan (66). The key implementation points were to establish a Road Traffic Injury Research and Prevention Center (RTIR&PC), a Road Crash Investigation system (involving traffic police, urban road network, insurance companies, and trauma registries), road safety, discipline, and compliance system, speed management, emergency services, and rehabilitation, improving motorcycling safety, implementing better licensing and helmet standards, and installing better road safety conditions for motorcyclists. However, this action plan was created in 2008 and there has been no updates in its implementation to date.

In order to improve the current situation of TBI treatment and care in Pakistan there is also a need to first create awareness about its importance in the society (56, 60). Strict measures should be taken to ensure the implementation of the traffic laws for e.g., to wear helmets. Number of neurosurgical centers, ambulances, and trained paramedics needs to increase (59). In addition to increase in neurosurgical centers, home-based caregivers must be trained to take care of TBI patients (67). To avoid long-term neurological and psychiatric disorders, regular checkups of the TBI patients after the injury can be done (57). This is where follow-up databases can really help.

Although we are limited by reporting no research data in the present review we hope our findings can be used to conduct a larger study in future. Our team has determined that Pakistan has an amalgamation of unique risk factors that lead to brain injuries such as lack of road legislations, terrorism (including suicide bombing), political instability, and domestic and sexual violence (34). Recent evidence further clarifies the molecular mechanisms underlying TBI that trigger amyloid precursor protein (APP) and Tau cleavage mediating AD pathology in animals (68). Additionally, the relationship of TBI and AD has shown to be quite complex and the presence of TBI leads to misdiagnoses of AD, interferes with treatment plans and makes research studies difficult to interpret (69). However, Pakistan lacks state-of-the-art diagnostic assessment of dementia and its risk factors, including neuroimaging of brain injury and aging that are extremely limited in Pakistan due to the inadequate infrastructure and limited training of clinicians (63). To help collaborative efforts, publications, and increasing awareness, we have established PBIC. The primary goal of PBIC will be to enhance collaborative efforts internationally and nationally through education, research, and publications on existing datasets. Specifically, we will teach emerging scientists to acquire and analyze data with high quality control and precision, and promote capacity building through training and educational endeavors. We believe that the large population in Pakistan provides a unique opportunity to yield high throughput research studies. Although low in numbers, a network of high-quality hospitals that are open to collaborate in developing local expertise in research and clinical care. In addition, Pakistan is one of the most philanthropic countries with substantial number of high net-worth individuals who can be invited to provide resources. Our ultimate goal is to bring Pakistan in the international arena for clinical neuroscience research and education and provide the data collected to global brain consortiums.

## Author Contributions

MA, MP, and AR contributed as primary authors including the concept, collaboration, literature search, writing first draft and editing. TS and MH provided literature searches, specifically those related to road traffic accidents in Pakistan. TS made the figures and tables. SS assisted with first draft of manuscript writing and editing. FM and SE provided literature feedback about brain injury numbers and databases that exist across various collaborating institutes and hospitals in Pakistan.

## Conflict of Interest

The authors declare that the research was conducted in the absence of any commercial or financial relationships that could be construed as a potential conflict of interest.
